# FDG-PET Quantification of Lung Inflammation with Image-Derived Blood Input Function in Mice

**DOI:** 10.1155/2011/356730

**Published:** 2011-12-10

**Authors:** Landon W. Locke, Mark B. Williams, Karen D. Fairchild, Min Zhong, Bijoy K. Kundu, Stuart S. Berr

**Affiliations:** ^1^Department of Biomedical Engineering, The University of Virginia, Charlottesville, VA 22908, USA; ^2^Department of Physics, The University of Virginia, Charlottesville, Virginia 22908, USA; ^3^Department of Radiology and Medical Imaging, The University of VA, Charlottesville, VA 22908, USA; ^4^Department of Pediatrics, The University of VA, Charlottesville, Virginia 22908, USA

## Abstract

Dynamic FDG-PET imaging was used to study inflammation in lungs of mice following administration of a virulent strain of *Klebsiella (K.) pneumoniae*. Net whole-lung FDG influx constant (*K*_*i*_) was determined in a compartment model using an image-derived blood input function. *Methods*. *K. pneumoniae* (~3 x 10^5^ CFU) was intratracheally administered to six mice with 6 other mice serving as controls. Dynamic FDG-PET and X-Ray CT scans were acquired 24 hr after *K. pneumoniae* administration. The experimental lung time activity curves were fitted to a 3-compartment FDG model to obtain *K*_*i*_. Following imaging, lungs were excised and immunohistochemistry analysis was done to assess the relative presence of neutrophils and macrophages. *Results*. Mean *K*_*i*_ for control and *K. pneumoniae* infected mice were (5.1 ± 1.2) ×10^−3^ versus (11.4 ± 2.0) ×10^−3^ min^−1^, respectively, revealing a 2.24 fold significant increase (*P* = 0.0003) in the rate of FDG uptake in the infected lung. Immunohistochemistry revealed that cellular lung infiltrate was almost exclusively neutrophils. Parametric *K*_*i*_ maps by Patlak analysis revealed heterogeneous inflammatory foci within infected lungs. *Conclusion*. The kinetics of FDG uptake in the lungs of mice can be noninvasively quantified by PET with a 3-compartment model approach based on an image-derived input function.

## 1. Introduction

The pulmonary inflammatory response is a highly coordinated multicellular process geared towards combating infection and injury. This response, which involves the activation and mobilization of a variety of cell types from the circulation to the site of injury, ultimately promotes the repair process. Although it is critical to survival, dysregulation of the inflammatory response can lead to persistent inflammation, which is believed to be a key factor in the pathogenesis of a wide variety of pulmonary diseases [1]. Neutrophils, a class of white blood cells, are often recruited to sites of infection. Because the presence of neutrophils and their secretory markers have been strongly linked to the pathophysiology of infections and tissue injury [2], there has been great effort aimed at understanding the role and behavior of these cells in the context of debilitating lung diseases.

Assessing neutrophil activation *in vivo *using the widely available [^18^F] fluoro-2-deoxyglucose (FDG) is an attractive technique for following inflammatory disease progression or the efficacy of novel anti-inflammatory drugs. As opposed to peptide-based imaging agents [3] or *ex vivo* laboratory labeling of white blood cells [4], FDG is well suited for tracking neutrophils due to their heightened glucose consumption required during reactive oxygen species generation [2, 5, 6]. This elevated demand for glucose forms the basis for probing neutrophil activation by FDG-PET. while FDG may not be the ideal inflammation tracer due to its sequestration in tumors as well as myocardial and brain tissue, it may be well suited to probe neutrophil metabolic activity due to the degree to which neutrophils consume it [2]. Prior* in vivo *studies have demonstrated neutrophil uptake of FDG in disease models ranging from animal models of pneumonia [7–9] to pancreatitis-induced lung injury [2] to sepsis-induced lung injury [6].

 Computing the net uptake rate of FDG by 3-compartmental model analysis of time-activity data is the current gold standard technique for quantitative measurement of glucose utilization. This technique requires an input function defined as the serial measurement of FDG activity concentration in the blood over the entire scan duration representing the quantity of tracer available to the tissues at each time point. There have been a variety of approaches taken to obtain the input function in mice including invasive arterial sampling, noninvasive estimation from dynamic PET images, and various combinations thereof [10–17]. We have shown in a prior publication that OSEM-MAP reconstruction with 18 iterations and cardiac gating eliminates spill-over (SP) contamination from the myocardium into the blood pool [18]. This allows for accurate measurement of the blood input function from the left ventricle on dynamic PET images with only minimal partial volume correction. As a consequence of the progress made in the derivation of the IDIF, there has been a renewed interest in the use of compartment models to quantify FDG uptake under inflammatory conditions in mice. The aim of this work is to establish the feasibility and accuracy of measuring FDG uptake to quantify bacteria-induced lung inflammation in mice using an input function that is noninvasively derived.

## 2. Materials and Methods

### 2.1. Experimental Animals

Twelve male C57BL/6 mice (8–10 weeks old, 25–30 g) were used in this study. The induction of bacterial pneumonia is based on a previously reported protocol [3]. Briefly, *K*. *pneumoniae* (strain 43816, serotype 2) was obtained from the American Type Culture Collection (Rockville, Md), grown overnight and then subcultured for 2 hours to log phase growth. After rinsing, bacteria were diluted in sterile normal saline for inoculation. Six mice were inoculated by oropharyngeal aspiration of 50 *μ*L of bacterial suspension (approximately 3 × 10^5^ CFU) under light inhalational anesthesia. Mice showed signs of moderate illness 18–36 hr after inoculation, when imaging was performed. Noninfected mice (*n* = 6) served as controls. The Animal Care and Use Committee of the University of Virginia approved all experiments.

### 2.2. PET and CT Protocol

Imaging was performed 24 hr following bacterial (or saline) induction. Following the insertion of a tail-vein catheter, ECG surface electrodes (Blue Sensor, Ambu Inc., Glen Burnie, MD) were placed on both forepaws and the left hindpaw. Anesthetized (1-2% isoflurane in oxygen) mice were placed in the prone position on a custom designed portable tray designed to reproducibly mount on both the CT and PET scanners, facilitating accurate image coregistration. Sixty-minute dynamic PET scans were performed using a Focus 120 PET scanner (Siemens Molecular Imaging Inc., Knoxville, TN). Data acquisition was initiated a few seconds before the injection of 18–20 MBq (486–540 *μ*Ci) FDG via the catheter. FDG was administered over the course of about 30 seconds and the catheter line was immediately flushed with 100 *μ*L of saline to clear the line and maximize FDG activity in the mouse. Physiological monitoring was performed with a Small Animal Instruments, Inc., model 1025L for PET (SAII, Inc., Stony Brook, NY). This device was used for monitoring heart rate, respiration, and core body temperature (rectal probe) while generating gate signals needed for retrospective reordering of time-stamped PET data into heart-cycle-based time bins. Following the PET scan, mice were immediately transported to the CT scanner [19], where a 10-minute whole-body CT scan was acquired for guiding lung ROI boundaries on PET images and correcting the PET data for attenuation [20]. All mice were fasted 4 hours prior to FDG administration.

### 2.3. Image Reconstruction

The PET reconstruction protocol is fully described in [18]. Briefly, list mode data were sorted into 23 dynamic frames and 3 cardiac-cycle time bins. PET images were reconstructed with attenuation correction using an OSEM-MAP algorithm [21] with 12 OSEM 3D subsets, 2 iterations, and 18 MAP iterations. 23 dynamic frames were generated for each cardiac bin. The frames consisted of eleven 8 s frames, one 12 s frame, two 1 min frames, one 3 min frame, and eight 6 min frames. The reconstructed image set was composed of 95 axial slices of thickness 0.79 mm with an in-plane voxel dimension of 0.4 mm × 0.4 mm (128 × 128 pixels).

CT images were reconstructed with a 3D filtered back-projection algorithm using COBRA software (Exxim, Inc., Pleasanton, CA). The CT reconstructed pixel size was 0.15 mm × 0.15 mm × 0.15 mm on a 320 × 320 × 384 image matrix but was downsampled to match the PET spatial resolution upon image coregistration. CT-based attenuation correction was performed using the PET scanner software.

### 2.4. Data Analysis

The net rate of FDG uptake in lung tissue was determined by fitting experimental PET-derived time-activity data to a 3-compartment model using nonlinear regression [22, 23]. Lung time-activity data was obtained as follows: ROIs outlining the lungs were manually drawn on CT images previously coregistered with PET data, where lung boundaries were clearly visible. All transverse slices containing lungs were analyzed for each mouse to cover the entire lung field. CT-drawn lung ROIs were then transferred to the PET images to obtain whole-lung tissue time-activity curves (TACs). The procedure for measuring the image-derived input function (IDIF) has been previously reported [18]. Briefly, IDIFs were measured by manually drawing small circular ROIs on contiguous transverse planes of the dynamic PET dataset in the area corresponding to the left ventricle (LV) blood pool and copying these ROIs on all frames. This process was repeated on two to three transverse slices in which the LV cavity was visible so that an average blood TAC could be computed. To correct for the partial volume effect in the LV blood pool, the IDIF was boosted by a predetermined recovery coefficient, as previously described [18]. 

 The net FDG influx constant (*K*_*i*_), which is linearly related to the local glucose metabolic rate, was computed in the lungs by kinetic analysis. Kinetic transport rate constants of FDG uptake in the lungs were estimated by the 3-compartment FDG model using nonlinear regression [23] by a program written in MATLAB (The Mathworks, Natick, MA, USA). From these estimated rate constants, whole-lung *K*_*i*_ values were computed.

To quantitatively assess the spatial distribution of FDG uptake within the lungs, selective slices from PET images were transformed into parametric images of local *K*_*i*_. Voxel-wise *K*_*i*_ values were computed by the Patlak analysis, which is based on the assumption that the ratio of tracer concentration in tissue to that in blood increases linearly when plotted as a function of the integrated blood radioactivity concentration normalized to the blood radioactivity measured at the midpoint of each frame [24]. To estimate *K*_*i*_, linear regression was performed on the Patlak plot beginning at 20 minutes after FDG injection to allow time for tracer equilibration between the blood and tissue compartments. 

### 2.5. Immunohistochemical Evaluation

Immunohistochemical (IHC) analysis was performed on harvested lung tissue to assess the relative distribution of neutrophils compared to macrophages in the lungs of control versus infected mice (25 hr after *K*. *pneumoniae* administration). Prior to lung removal, the pulmonary circulation was flushed with saline via the right ventricle to eliminate nonadherent white blood cells. Lungs were then inflated with formalin to distend the alveolar spaces uniformly. The trachea was cannulated and 10% phosphate-buffered formalin infused at a pressure of 25 cm H_2_O. After fixation, the lung was dissected coronally in the plane of the mainstem bronchus. Adjacent histological sections (3 *μ*m thick) were specifically stained for either neutrophils with a monoclonal rat anti-mouse neutrophil IgG (MCA771G; Serotec) or for macrophages with anti-MAC-2 IgG (ACL8942P; Accurate). Stained cells were observed under a light microscope (Microphot, Nikon, LRI Instruments AB, Tokyo, Japan).

### 2.6. Statistical Analysis

Group data are expressed as the mean ± standard deviation. Student's *t*-test was used to compare results among groups. Statistical significance was set at *P* < 0.05. Sigma-Stat v2.0 (SPSS, Inc, Chicago, IL) was used for statistical testing.

## 3. Results

### 3.1. Compartmental Modeling and Whole-Lung *K*_*i*_

**Figure 1:**
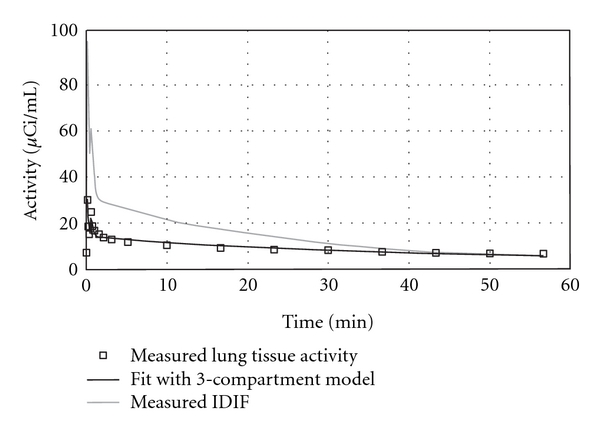
A representative plot showing a lung tissue TAC from a control animal and the associated compartment model fit (R^2^= 0.92). Modeling produced good fits to the observed data.

**Figure 2:**
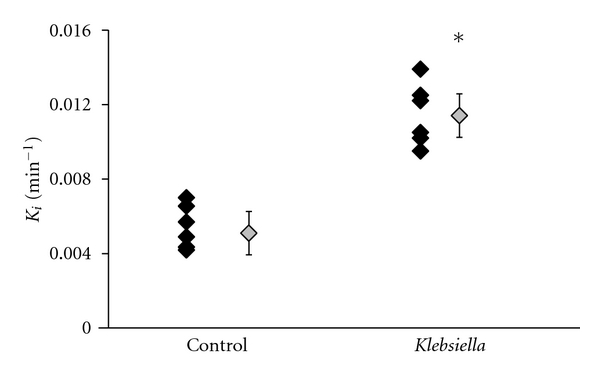
Whole-lung *K*_*i*_ values for control and *K*. *pneumoniae-*challenged mice calculated from 3-compartment model analysis (see Section 2). The black diamonds represent lung *K*_*i*_ values of individual mice while the gray diamonds represent the average of the group. *P < 0.05 compared with the noninfected control group.

An example of a lung TAC and the associated compartmental model fit from a control animal is shown in Figure 1. The figure demonstrates that the model output fits the experimental lung data very well. Whole-lung FDG influx constant (*K*_*i*_) was significantly higher in the group of mice administered *K*. *pneumoniae* compared to controls. The mean whole-lung *K*_*i*_ for control mice was (5.1 ± 1.2) × 10^−3^ versus (11.4 ± 2.0) × 10^−3^ min^−1^ for the *K*. *pneumoniae*-challenged mice (*P* = 0.0003) shown as gray diamonds in Figure 2. Within each group, individual whole-lung lung *K*_*i*_ values are shown (black diamonds).

### 3.2. Parametric Lung Maps

**Figure 3:**
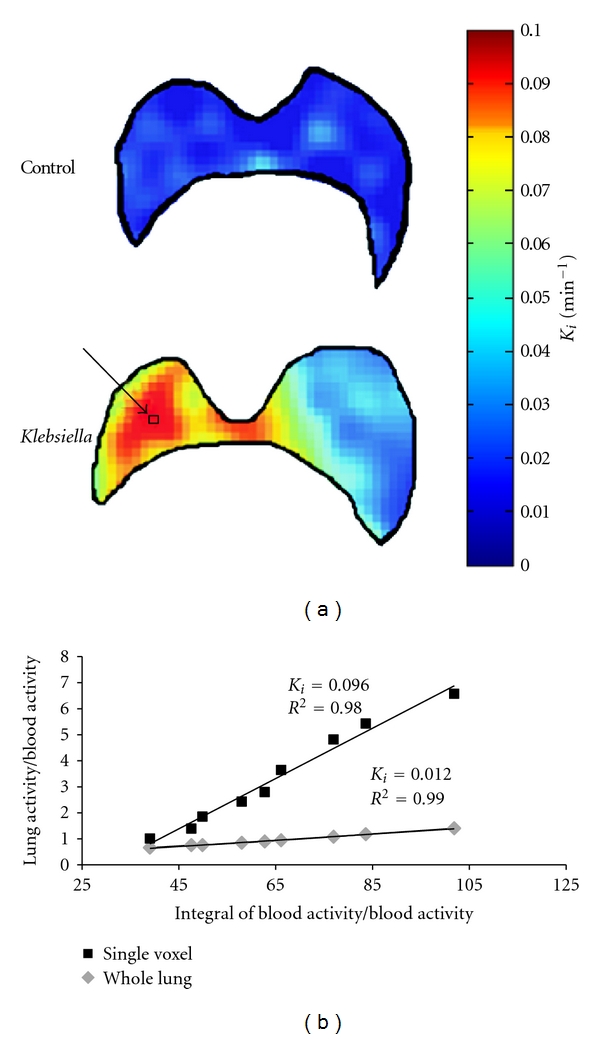
Parametric *K*_*i*_ images of segmented lungs of a control mouse and *K*. *pneumoniae*-challenged mouse computed by the Patlak analysis (a). The *K*_*i*_ value of a single voxel within an area of high FDG uptake (indicated by arrow) is significantly higher than the whole-lung *K*_*i*_ value computed by the Patlak method (b), leading to the skewing effect observed between the apparent average lung *K*_*i*_ of the slice and the whole-lung *K*_*i*_ value estimated from the three-compartment model analysis.

Representative parametric *K*_*i*_ maps of lungs from a control mouse and a *K*. *pneumoniae*-challenged mouse are shown in Figure 3(a). Voxel-wise *K*_*i*_ values were computed by the Patlak analysis using the image-derived blood input function. Segmented lung boundaries were identified and manually outlined on coregistered CT images. Hyperintense regions are clearly visualized on the *K*_*i*_ parametric maps of the *K*. *pneumonia-*infected mice. The *K*.* pneumoniae*-challenged mouse clearly demonstrates the spatially heterogeneous nature of the lung disease model. 

### 3.3. Immunohistochemistry

**Figure 4:**
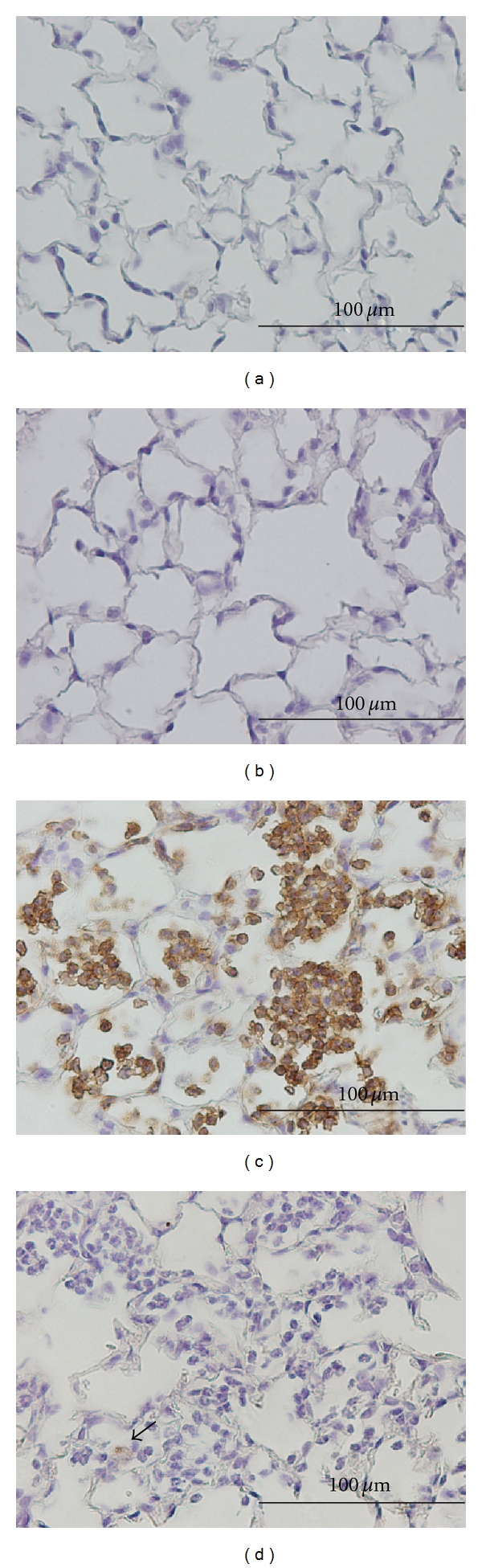
Immunohistochemical staining of neutrophils (stained with a rat anti-mouse IgG) and macrophages (stained with anti-MAC-2 IgG) in lung tissue excised from a control and a *K*. *pneumonia-*infected mouse (42 hr after challenge). Immunostained cells appear dark brown. Control lungs revealed no neutrophils (a) or macrophages (b), only normal alveolar wall structure. Infected lungs had significant neutrophil accumulation (c), with very little macrophage infiltration (d, indicated by arrowheads). Images were taken with a magnification of 400x, black bar indicates 100 *μ*m.

Immunohistochemical staining of lung tissue from a control mouse revealed normal alveolar wall structure with very few infiltrated neutrophils (Figure 4(a)) or macrophages (Figure 4(b)). In contrast, the *K*. *pneumonia*-infected mouse (euthanized 25 hr post *K*. *pneumoniae *challenge) had significant lung neutrophil burden (Figure 4(c)), with very little macrophage infiltration (Figure 4(d)), as indicated by arrowheads.

## 4. Discussion

Compartmental modeling is considered the gold standard technique for quantifying FDG uptake and may provide a better representation of disease activity than static measurements, which can fluctuate with time after-injection. In this work we established the means to quantitatively assess pulmonary ^18^F-FDG uptake in mice by compartmental modeling using an IDIF. Quantitative determination of kinetic parameters can be made either at a regional level or at the voxel level. Because many lung diseases can be spatially heterogeneous, it may be beneficial to transform PET images into parametric maps of local *K*_*i*_. Disease processes that are not distinguishable by visual inspection of FDG PET images (e.g., inflammation and cancer) may be distinguishable using the quantitative parametric maps. The drawback of voxel-wise analysis is the increased amount of noise of voxel TACs compared to ROI TACs. This may lead to inaccuracies in the numerical identification of the kinetic parameters being estimated when fitting data to a compartmental model. We found that the Patlak analysis is better suited to estimate *K*_*i*_ at the voxel level because it relies on a simple graphical transformation of the time-activity data to directly compute *K*_*i*_. 

The higher coefficient of variance (CoV) in the mean *K*_*i*_ of the control group compared to the group challenged with *K*. *pneumoniae *(23% versus 17%) is a result of a 4% larger CoV in control lung TACs compared to the lung TACs measured from the *K*. *pneumoniae*-challenged group. However, there was no difference in the CoV of IDIFs between the two groups. The CoV and mean whole-lung *K*_*i*_ computed here for the control group are in close agreement with that which was reported by Zhou et al. ((5.9 ± 1.0) × 10^−3^) for mice of similar age and background strain derived from arterial-based blood sampling.

There appears to be an apparent discrepancy between whole-lung *K*_*i*_ values estimated from the three-compartment model and voxel-wise *K*_*i*_ values shown in Figure 3(a) computed by the Patlak analysis for *K*. *pneumoniae*-challenged mice. This can be attributed to the fact that the Patlak analysis was done on select axial lung slices that best highlighted *K*. *pneumoniae*-induced inflammation, thus skewing the apparent average lung *K*_*i*_ value compared to whole-lung *K*_*i*_ values estimated from the three-compartment model. This is demonstrated in Figure 3(b) which shows the result of the Patlak analysis performed on a single voxel (indicated by arrow) and all lung field voxels. The *K*_*i*_ value computed for the single voxel corresponding to an area of intense FDG uptake is significantly elevated compared to the Patlak-derived whole-lung *K*_*i*_, leading to the skewing effect described above. The average *K*_*i*_ value for the control lung (Figure 2) derived from multiple slices averaged over six mice is comparable to the average that can be inferred from a single slice in the voxel-wise Patlak plot (Figure 3(a)). 

There have been studies designed to prove the specificity of the FDG imaging signal for metabolically active neutrophils in the lung. One such study examined mice chemically depleted of neutrophils and the resulting effect on lung FDG uptake following an inflammatory challenge [9]. Results from this study showed that when neutrophils are depleted the level of FDG sequestration and myeloperoxidase activity is reduced in the endotoxin-challenged lung. This is consistent with the hypothesis that neutrophils, likely due to their increased metabolic demand during respiratory burst, are the dominant cell type responsible for FDG uptake. Our immunohistochemical analysis, which revealed that neutrophils are the predominant cell infiltrating the lung interstitium and alveoli at the time point studied, supports this hypothesis.

## 5. Conclusion

The novelty of this technique lies in its noninvasive approach in modeling pulmonary FDG uptake in mice. Using three-compartment modeling based on an input function that is image-derived, we measured a 2.24-fold increase in FDG uptake (*K*_*i*_) in the lungs of *K*. *pneumoniae*-challenged mice compared to noninfected controls. Furthermore, our computed mean lung *K*_*i*_ values were not significantly different from previously reported values for mice of similar age and background strain computed from input functions obtained by arterial sampling.

Transforming dynamic PET data into parametric maps of local *K*_*i*_ via the Patlak analysis may be a more informative technique for studying lung diseases that are spatially heterogeneous or for assessing local response to therapy. This completely noninvasive approach may widen the accessibility of this technique for monitoring inflammatory lung diseases and therapies targeting neutrophilic inflammation.
